# Some pharmacokinetic parameters of salvianolic acid A following single-dose oral administration to rats

**DOI:** 10.1080/13880209.2018.1491998

**Published:** 2018-08-19

**Authors:** Jialin Sun, Junke Song, Wen Zhang, Fanbo Jing, Wen Xu, Ping Leng, Xianghua Quan, Guanhua Du, Zhongguo Sui

**Affiliations:** a Department of Pharmacy, The Affiliated Hospital of Qingdao University, Qingdao, People’s Republic of China;; b Beijing Key Laboratory of Drug Target Identification and Drug Screening, Institute of Materia Medica, Chinese Academy of Medical Sciences & Peking Union Medical College, Beijing, People’s Republic of China

**Keywords:** Absorption, bioavailability, transportation, first-pass metabolism, elimination

## Abstract

**Context:** Salvianolic acid A (Sal A) is a hydrophilic bioactive compound isolated from *Salvia miltiorrhiza* Bunge (Lamiaceae). It exerts beneficial effects after oral administration on diabetic complications.

**Objective:** To systematically study the absorption, distribution and excretion of Sal A after single-dose oral administration.

**Materials and methods:** Animal experiments were conducted in Sprague-Dawley rats. Plasma was sampled at designated times after oral doses of 5, 10 and 20 mg/kg, and an intravenous dose of 50 μg/kg. Tissues were harvested at 10, 60 and 120 min postdosing. Bile, urine and feces were collected at specified intervals before and after dosing. Absorption and distribution characteristics were analyzed by LC–MS, and excretion characteristics were analyzed by UPLC–MS/MS. The Caco-2 cell model was applied to investigate potential mechanisms.

**Results:** The *C*_max_ (5 mg/kg: 31.53 μg/L; 10 mg/kg: 57.39 μg/L; 20 mg/kg: 111.91 μg/L) of Sal A increased linearly with doses (*r*> 0.99). The calculated absolute bioavailability was 0.39–0.52%. Transport experiment showed poor permeability and the ratio of *P*_B–A_ to *P*_A–B_ was 3.13–3.97. The highest concentration of Sal A was achieved in stomach followed by small intestine and liver, and it could also be detected in brain homogenate. Approximately 0.775% of its administered dose was excreted via feces, followed by bile (0.00373%) and urine (0.00252%).

**Discussion and conclusions:** These results support the future development of Sal A as an oral drug for the treatment of diabetic complications. Future research should be conducted to investigate the reason for its poor bioavailability and improve this situation.

## Introduction

**Figure 1.**
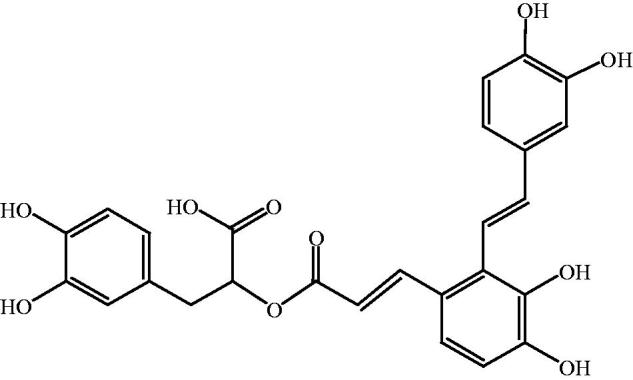
Chemical structure of Sal A.

Traditional Chinese medicines (TCMs) are natural therapeutic agents used according to the guiding theory of traditional Chinese medical science. Danshen, the dry root or rhizome of *Salvia miltiorrhiza* Bunge (Lamiaceae), has been used for thousands of years for the treatment of cardiocerebral vascular diseases (Huang et al. 2016), chronic renal failure (Cai et al. 2018), retinopathy (Zhang et al. 2013) and liver dysfunction (Xu et al. 2013). Salvianolic acid A (Sal A) is the main hydrophilic bioactive compound of Danshen. The chemical structure of Sal A is shown in Figure 1. Pharmacological studies found that Sal A exerted various therapeutic activities after intravenous administration, such as cardiac protection against ischemia reperfusion injury (Chen et al. 2016), inhibition of platelet aggregation (Yuan et al. 2017), reduction of oxygen radicals’ release (Gu et al. 2017), and inhibition of tumor growth (Zheng et al. 2015).

Some recent research showed that the administration route of Sal A was not limited to intravenous administration. It could also play a therapeutic effect after oral administration, such as the protective function on diabetic complications including beneficial effects on plantar microcirculation and peripheral neurological dysfunction (Yang et al. 2011b; Yu et al. 2012; Hou et al. 2017), and protective effect against diabetic vascular endothelial dysfunction (Yang et al. 2011a).

In order to promote the development of Sal A as a potential oral drug for the treatment of diabetic complications, it is necessary to systematically study the metabolic characteristics of Sal A monomer, including absorption, distribution and excretion. The intravenous pharmacokinetics of Sal A has been reported in many articles, using either Sal A alone or as a part of an herbal mixture (Zhao et al. 2011; Wang et al. 2013; Feng et al. 2017; Wang et al. 2017). In contrast, oral pharmacokinetic studies of this compound were rare.

Currently, there are only two reports regarding the pharmacokinetics of Sal A after oral administration in pure form; one in Sprague-Dawley rats (Pei et al. 2008) and the other in Beagle dogs (Sun et al. 2013). Absolute bioavailability of the compound was only reported in the latter one, which was calculated to be 1.25% in dogs. Another report concerning the bioavailability of Sal A studied the pharmacokinetics of salvianolic acid extract at a high oral dose of 800 mg/kg in rats (Lai et al. 2011). The mixture mainly consisted of salvianolic acid B (50.6%), rosmarinic acid (3.92%) and Sal A (5.71%). The absolute bioavailability of Sal A was calculated to be 2.5%, twice the value in dogs. Drug interactions tend to exist when drugs are used in combination, and pharmacokinetic interactions were observed among the components of *Salvia miltiorrhiza* extract (Chang et al. 2010). Based on the above studies, some questions were raised. Is the numerical difference caused by species differences or drug interactions? In addition, can Sal A reach an effective concentration after oral administration under such low bioavailability? To figure out the answers, it is necessary to study the metabolic dynamics of Sal A after oral administration in rats in pure form.

Besides the absorption studies, the distribution and excretion characteristics of Sal A have not been reported so far. Systematically studying the oral pharmacokinetic behavior of Sal A in rats, including absorption and the related mechanisms, distribution and excretion, is of great value in promoting its subsequent development.

## Materials and methods

### Chemicals and reagents

Sal A was isolated from the roots of *Salvia miltiorrhiza* by our laboratory. Raw material was collected in October 2009; the plant identification was conducted by associate professor Lin Ma of the Institute of Materia Medica, Chinese Academy of Medical Sciences, Beijing, China. A voucher specimen (No. NCPS008) was deposited in the laboratory for further reference. Taking the dried roots of *Salvia miltiorrhiza* as raw material, Sal A was isolated after crushing, solvent extraction, macroporous resin column separation, concentration, and drying processes, with a purity of over 94% by HPLC analysis (Du et al. 2012).

Butyl paraben (internal standard, IS) was obtained from the National Institutes for Food and Drug Control (Beijing, China). Caco-2 cells were bought from American Type Culture Collection (VA, USA). Acetonitrile (CH_3_CN, LC–MS-grade) and methanol (MeOH, LC–MS-grade) were purchased from J.T. Baker (Seattle, WA). Formic acid (HCOOH, HPLC-grade) was obtained from TEDIA (Fairfield). All the other reagents were of analytical grade. Hydrochloric acid (HCl) and ethyl acetate (EtOAc) were purchased from Beijing Chemical Reagent Co. (Beijing, China). Ascorbic acid (Vitamin C, VC) was from Sigma-Aldrich (St. Louis, MO). Minimal essential medium nonessential amino acid (MEM NEAA), sodium pyruvate, penicillin–streptomycin and HEPES were obtained from GIBCO (Langley, OK). Transwell plates were obtained from CORNING (Corning, NY). Anticoagulation tubes with heparin were from Jiangsu Kangjian Healthcare Co. Ltd (Taizhou, Jiangsu, China). Watsons distilled water (Hongkong, China) was used throughout the study.

### Instrumentations and conditions

An Agilent 1200 liquid chromatography-6110 mass spectrometer (California) was used to determine Sal A’s concentration in plasma, Hank’s Balanced Salt Solution (HBSS) and tissue samples by our reported LC–MS method (Sun et al. 2013) in negative ion mode.

Typical parameters for mass spectrum were set as follows: the spray chamber parameters, including capillary voltage, nebulizer pressure, drying gas flow rate and drying gas temperature were –3,000 V, 35 psig, 10 L/min and 350 °C, respectively; the compound parameters, including frag mentor, gain and dwell time were –75 V, 1.5 and 144 ms for both Sal A and IS, respectively. Target ions were monitored at *m*/*z* 493 for Sal A and 193 for IS.

LC separations were performed on a 3.5 μm Agilent Zorbax SB-C_18_ (100 × 2.1 mm, i.d.) at 30 °C with an Agilent Zorbax SB-C_18_ guard column used before the analytical column. The mobile phase, which was mixed with solvent A (H_2_O, containing 0.05% HCOOH) and solvent B (CH_3_CN, containing 0.05% HCOOH), was gradient elution at a flow rate of 0.3 mL/min. Initial condition of the gradient program was solvent A–B (85:15, v/v) and changed to A–B (25:75, v/v) in 5 min, then returned to initial state at 5.01 min. Total running time was 10 min. The injection volume was 20 μL.

Due to the complex components and interference in the excretion samples, an UPLC–MS/MS method with a better separation and a higher resolution was applied to quantify the content of Sal A in urine, bile and feces samples. The UPLC–MS/MS system consisted of a Waters Acquity UPLC (MA) coupled to an AB ACIEX API 4000 triple-quadrupole mass spectrometer (Boston) equipped with a turbo ion spray ion source.

Typical parameters used in this method were set as follows: the source parameters, including curtain gas, gas 1, gas 2, collision gas (CAD), capillary temperature (TEM) and ion spray voltage, were set at 20, 40, 40, 6 psi, 500 °C and –4,000 V, respectively; the compound parameters, including declustering potential (DP), collision energy (CE), entrance potential (EP) and collision cell exit potential (CXP), were –70, –22, –10 and –10 V for both Sal A and IS, respectively. The MS detector was operated by multiple-reaction monitoring (MRM) method in the negative ion mode. The precursor-to-product ion transition for Sal A was *m*/*z* 493 → 295, and for IS was *m*/*z* 193 → 137.

LC separations were performed on a 1.7 μm ACQUITY UPLC BEH C_18_ column (50 × 2.1 mm, i.d.) at room temperature. The mobile phase, which was mixed with solvent A (H_2_O, containing 0.1% HCOOH) and solvent B (CH_3_CN, containing 0.1% HCOOH), was gradient elution at a flow rate of 0.5 mL/min. Initial condition of the gradient program was solvent A–B (90:10, v/v), kept for 0.5 min, changed to A–B (76:24, v/v) in 9.5 min, changed to A–B (10:90, v/v) at 10.2 min, and kept for 1 min, then returned to initial state at 11.5 min. Total running time was 12 min. The injection volume was 5 μL.

### Pharmacokinetic study in vivo

#### Animals

Ninety-six healthy, intact Sprague–Dawley rats (male/female, 1/1, 185–215 g, Certificate No. SCXK 2006-0009) were purchased from Vital River Laboratories (Beijing, China). The rats were maintained in air-conditioned animal quarters with alternating 12 h light/dark cycles at a room temperature of 22 ± 2 °C and a relative humidity of 50 ± 10%. The rodents were given a commercial rat chow and water *ad libitum*. The experiment started after acclimation to the facilities for 7 days. All animal studies were conducted in accordance with “Principles of laboratory animal care” (NIH publication No. 85-23, revised 1985) and under protocols approved by the Ethic Committee of Laboratory Animals of the Affiliated Hospital of Qingdao University (QYFYKYLL 2013-2-4-01).

#### Experimental design

Rats were fasted overnight (∼12 h) and had free access to water throughout the experimental period. In our study, rats were treated as follows: 24 rats (male/female, 1/1) were given a single dose of Sal A at 50 μg/kg by intravenous injection; the remaining 72 rats were divided into three groups randomly (24 rats/group, male/female, 1/1) and were given a single oral dose of 5, 10 and 20 mg/kg, respectively. The doses applied in this study were determined according to, on the one hand, the doses applied in pharmacodynamic experiments in rats (Yang et al. 2011a; Yang et al. 2011b; Yu et al. 2012; Hou et al. 2017), and on the other hand, the results of our preliminary absorption experiments. The blood samples were taken crossly from the orbital sinus into tubes pretreated with heparin before dosing and subsequently at 0.083, 0.167, 0.25, 0.333, 0.5, 0.75, 1, 2, 4, 8, 12 and 24 h postdosing. After blood sampling, the samples were centrifuged at 4,500 rpm for 15 min at 4 °C to obtain 200 μL of plasma samples. These samples were immediately frozen and maintained at –80 °C until analysis.

#### Sample preparation

Plasma samples were thawed at room temperature before processing. To a 200 μL aliquot of plasma sample, 10 μL of VC (10 mg/mL) and 10 μL of the IS spiking solution (butyl paraben, 1 μg/mL) were added. Then, the mixture was acidified with 100 μL of 1 mol/L HCl and vortex-mixed with 800 μL of EtOAc for 3 min. After centrifugation at 13,400 rpm for 10 min, the upper organic layer was transferred to a clean Eppendorf tube. EtOAc (800 μL) was added again to the lower aqueous phase and the extraction process was repeated. The two upper organic layers were combined and evaporated to dryness under nitrogen at 30 °C. The residue was reconstituted in 100 μL of MeOH–H_2_O (50:50, v/v, containing VC 100 μg/mL). After centrifugation at 13,400 rpm for 5 min, 20 μL of the supernatant was injected to the LC–MS system.

#### Pharmacokinetics analysis

DAS pharmacokinetic software Data Analysis System Version 3.0 (Bontz Inc., Beijing, China) was used for pharmacokinetic analysis of Sal A concentrations. The noncompartmental model was applied for analysis of the data following intravenous and oral administration of Sal A. The peak plasma concentration (*C*_max_), the corresponding time (*T*_max_), the mean residence time (MRT) and the area under the concentration–time curve (AUC) could all be obtained.

### Permeability study in Caco-2 monolayers

#### Cell culture

Caco-2 cells (passage number 34–36, American Type Culture Collection, Manassas, VA) were cultured at 37 °C in an atmosphere of 5% CO_2_ and 90% relative humidity in Dulbecco’s modified Eagle’s medium supplemented with 10% fetal bovine serum, 1% penicillin–streptomycin, 1% HEPES, 1% sodium pyruvate and 1% minimal essential medium nonessential amino acids. After harvesting at 90% confluence, the cells were seeded on to 0.4 μm polycarbonate membrane transwell inserts (12 mm diameter) at a density of 1.5 × 10^5^ cells/cm^2^. The culture media was changed every other day during the first 7 days after seeding and once a day thereafter. Cell monolayers were ready for experiments from 20 to 24 days postseeding.

#### Transport experiments in the Caco-2 cell culture model

Experiments in triplicate were performed in HBSS (pH =7.4).To ensure the stability of Sal A in HBSS, VC was applied in this study as stabilizer. The monolayers were washed three times with prewarmed HBSS (37 °C) before use. The integrity of the Caco-2 monolayers was monitored by measuring the transepithelial electrical resistance, and only monolayers with values ≥400 Ω/cm^2^ were used. The monolayers were incubated with HBSS at 37 °C for 30 min, and then the incubation medium was aspirated. Afterwards, bidirectional transport experiments were conducted. Sal A solution of appropriate concentrations in HBSS (containing VC 1 0^−4^ mol/L) were loaded to the donor compartments and the blank HBSS (containing VC 1 0^−4^ mol/L) was added to the receiver compartments. Seven sequential samples (50 μL) were collected at different times (0, 30, 60, 90, 120, 150, 180 min) from the receiver side. The same volume of receiver medium was replaced after each sampling. The collected samples were added into tubes with 40 μL MeOH–H_2_O (50:50, v/v, containing VC 100 μg/mL). Then, 10 μL of the IS spiking solution (butyl paraben, 1 μg/mL) was added. After centrifugation at 13,400 rpm for 5 min, 20 μL of the supernatant was injected to the LC–MS system.

Influence of the stabilizer on the transport of Sal A has also been tested under the condition of different concentrations of VC. The samples were collected, treated and analyzed according to the same procedure.

#### Data analysis

The apparent permeability coefficient (*P*_app_) was calculated according to the following equation:

$$P_{\text{app}}=\left(\Delta Q/\Delta t\right)/\left(A\times C_{0}\right)$$

where Δ*Q*/Δ*t* is the linear appearance rate of the tested compound in the receiver compartments, *A* is the surface area of the cell monolayer and *C*_0_ is the initial concentration of the tested compound in the donor side.

### Tissue distribution of Sal A in rats

#### Animals

Twenty-four healthy, intact Sprague–Dawley rats (male/female, 1/1, 185–215 g, Certificate No. SCXK 2006-0009) were purchased from Vital River Laboratories (Beijing, China). The rats were maintained in air-conditioned animal quarters with alternating 12 h light/dark cycles at a room temperature of 22 ± 2 °C and a relative humidity of 50 ± 10%. The rodents were given a commercial rat chow and water *ad libitum*. The experiment started after acclimation to the facilities for 7 days. The animal protocols used in this study followed guidelines set forth by the Ethic Committee of Laboratory Animals of the Affiliated Hospital of Qingdao University (QYFYKYLL 2013-2-4-01).

#### Experimental design

Rats were fasted overnight (∼12 h) and had free access to water throughout the experimental period. In our study, rats were treated as followed: 18 rats were divided into 3 groups randomly (6 rats/group, male/female =1/1) and were all given a single oral dose of 5 mg/kg. The animals were sacrificed 10 min, 1 and 2 h postdosing and stomach, small intestine, heart, liver, spleen, lung, kidney and brain were harvested for drug analysis. The remaining six rats were given normal saline as control. Tissue samples were frozen and maintained at –80 °C immediately until analysis.

#### Sample preparation

Tissues were homogenized in normal saline (2 mL/g, except 6 mL/g for stomach) using a FLUKO F6/10 for 1–2 min over an ice bath. The aliquots of 200 μL homogenized tissues were used for the LC–MS analysis. The remaining steps were the same as sample preparation in “Pharmacokinetic study *in vivo*”.

### Excretion of Sal A from rats

#### Animals

Twelve healthy, intact Sprague–Dawley rats (male/female, 1/1, 185–215 g, Certificate No. SCXK 2006-0009) were purchased from Vital River Laboratories (Beijing, China). The rats were maintained in air-conditioned animal quarters with alternating 12 h light/dark cycles at a room temperature of 22 ± 2 °C and a relative humidity of 50 ± 10%. The rodents were given a commercial rat chow and water *ad libitum*. The experiment started after acclimation to the facilities for 7 days. The studies were conducted under protocols approved by the Ethics Committee of Laboratory Animals of the Affiliated Hospital of Qingdao University (QYFYKYLL 2013-2-4-01).

#### Experimental design

In the urine and feces excretion studies, rats were treated as follows. After acclimation to the facilities for 7 days in metabolism cages and being fasted overnight (∼12 h) with free access to water, six rats (male/female, 1/1) were given a single dose of Sal A at 20 mg/kg orally. Blank feces and urine were got prior to administration. Feces samples were collected from 0 to 12, 12 to 24, 24 to 48, 48 to 72 and 72 to 96 h. Urine samples were collected from 0 to 6, 6 to 12, 12 to 24, 24 to 48, 48 to 72 and 72 to 96 h. These samples were immediately frozen and maintained at –80 °C until analysis.

In the bile excretion study, rats were treated as follows. After acclimation to the facilities for 7 days and being fasted overnight (∼12 h) with free access to water, six rats (male/female, 1/1) received a bile duct intubation surgery. An oral dose of 20 mg/kg were given to the postoperative awake rats, and bile samples were collected prior and after the administration (from 0 to 2, 2 to 4, 4 to 6, 6 to 8, 8 to 12 and 12 to 24 h). The samples were immediately frozen and maintained at –80 °C immediately until analysis.

#### Sample preparation

After drying, feces were soaked in purified water containing VC 100 μg/mL (5 mL/g) overnight and treated with ultrasonic for 10 min. Homogenate was centrifuged at 13,400 rpm for 10 min. 200 μL of the supernatant was got for the UPLC–MS/MS analysis, along with 200 μL of urine and bile samples. Sample preparation was the same as sample preparation in “Pharmacokinetic study *in vivo*”.

### Statistical analysis

The data in this paper were presented as mean ± SEM, if no specified otherwise. Significant differences were assessed using unpaired Student’s *t* test. A *p* value of <0.05 was considered as statistically significant.

## Results

### Pharmacokinetic studies

**Figure 2.**
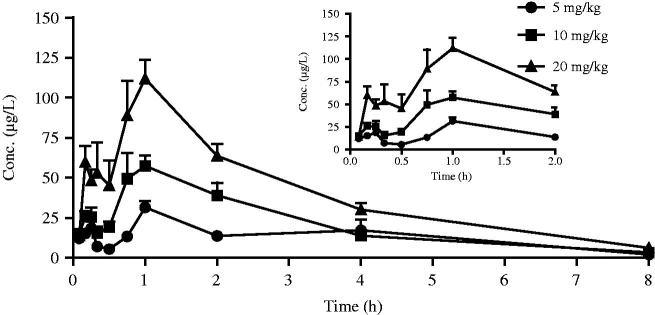
The mean plasma concentration–time curves of Sal A following single oral doses of 5, 10 and 20 mg/kg to rats (*n*= 12).

**Figure 3.**
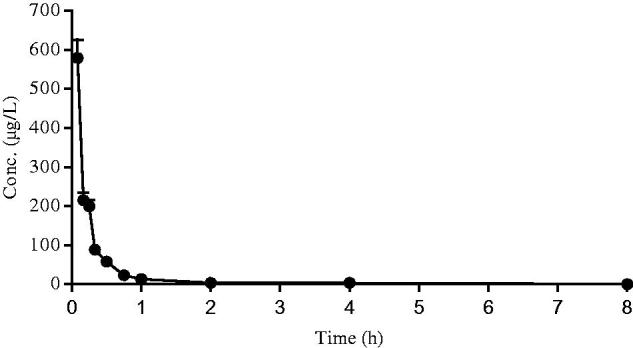
The mean plasma concentration–time curve of Sal A following a single intravenous dose of 50 µg/kg to rats (*n*= 12).

After intravenous and oral administration of Sal A to rats, plasma concentration was determined by LC–MS. The data obtained from each group was averaged. Figures 2 and 3 showed the mean plasma concentration–time curves of Sal A in rats receiving oral doses of 5, 10 and 20 mg/kg and an intravenous dose of 50 µg/kg, respectively. The pharmacokinetic parameters derived from plasma concentration–time profiles were summarized in Table 1. The oral bioavailability (*F*) was calculated by using AUC_oral_/dose divided by AUC_iv_/dose.

**Table 1.** Pharmacokinetics parameters of Sal A in rats (*n*= 12).

Parameters	Unit	Oral			i.v.
		5 mg/kg	10 mg/kg	20 mg/kg	50 µg/kg
AUC_(0–_*_t_*_)_	μg/L h	105.93	167.18	317.11	204.57
*C*_max_	μg/L	31.53	57.39	111.91	578.89
T_max_	H	1.00	1.00	1.00	0.08
t_1/2_	H	1.96	1.72	1.79	6.16
MRT_0–t_	H	2.91	2.33	2.36	0.62
F	%	0.52	0.41	0.39	–

After oral administration, Sal A reached its peak concentration in plasma within 1 h, indicating that this phenolic acid was readily absorbed. Plasma levels of Sal A decreased rapidly after dosing, and the calculated half-life period (*t*_1/2_) was 1.72–1.96 h, which was shorter than that observed after intravenous administration (6.16 h). The *C*_max_ values were estimated to be 31.53, 57.39 and 111.91 µg/L, respectively. The AUC increased with increasing doses, and the AUC_(0–_*_t_*_)_ values were 105.93, 167.18 and 317.11 (µg/L h), respectively. The good linearity of the kinetics of Sal A after oral administration was observed in the regression analysis of the AUC–dose plot (*r*> 0.99) and the *C*_max_*–*dose plot (*r*> 0.99). The calculated oral bioavailability was quite low, ranging from 0.39 to 0.52%.

### Transport of Sal A across Caco-2 cell monolayer

The Caco-2 transport model, which was recognized by FDA, has been widely used to classify the absorption characteristics of a compound (Lu et al. 2008; Yang et al. 2010). Table 2 summarized the *P*_app_ values for different concentrations of Sal A (1 0^−4^/27, 1 0^−4^/9 and 1 0^−4^/3 mol/L) from the apical to basolateral and basolateral to apical sides of the Caco-2 cell model. Influence of VC to the bidirectional transport was investigated and the results were shown in Table 3.

**Table 2.** Apparent permeability for different concentrations of Sal A in Caco-2 cell monolayer (*n*= 3).

	*P*_app_×10^–7^ (cm/s)	
Conc. (mol/L)	Apical → basolateral	Basolateral → apical
10^–4^/27	6.407 ± 0.576	9.866 ± 1.212
10^–4^/9	1.525 ± 0.055	6.050 ± 0.175
10^–4^/3	1.564 ± 0.412	5.081 ± 0.118

**Table 3.** Apparent permeability for Sal A (10^–4^/9 mol/L) under the conditions of different VC concentrations in Caco-2 cell monolayer (*n*= 3).

	*P*_app_×10^–7^ (cm/s)	
Conc. (mol/L)	Apical → basolateral	Basolateral → apical
10^–4^	1.525 ± 0.055	6.050 ± 0.175
10^–3^/3	1.548 ± 0.030	5.069 ± 0.870
10^–3^	1.528 ± 0.028	4.781 ± 0.453

A compound might be absorbed poorly, moderately or well with a *P*_app_ value of <1 × 1 0^−6^, 1 × 1 0^−6^–10 × 1 0^−6^, or >10 × 1 0^−6^ cm/s, respectively. Accordingly, the measured *P*_app_ value of Sal A was 1 × 1 0^−7^–7 × 1 0^−7^ cm/s (<1 × 1 0^−6^ cm/s), suggesting a poor membrane permeability. The ratio of the *P*_app_ values of basolateral to apical and apical to basolateral were about three in our experiment, indicating that the transport of Sal A across the Caco-2 monolayer involved an active transport mechanism.

Due to the instability of Sal A in the partial neutral environment of Hanks, we have applied VC as a stabilizer, and the impact of VC to the results were shown in Table 3. Statistical analysis showed that there was no significant difference in the amount of Sal A transported across the Caco-2 monolayer at different VC concentrations (*p*> 0.05), which meant that VC did not affect the transportation of Sal A.

### Tissue distribution of Sal A in rats

**Figure 4.**
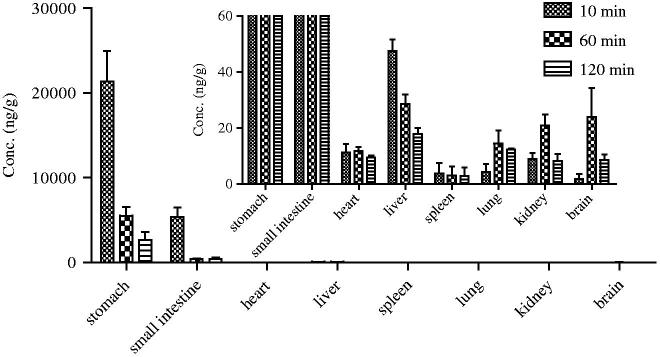
Tissue distribution of Sal A at 10, 60 and 120 min following a single-dose oral administration of 5 mg/kg to rats (*n*= 6).

The tissue distribution of Sal A after a single oral dose of 5 mg/kg in rats at 10 min, 1 and 2 h was presented in Figure 4. Results showed that Sal A could distribute widely in various organs *in vivo*, including the stomach, small intestine, heart, liver, spleen, lung, kidney and brain. Furthermore, Sal A was found to distribute into tissues within 10 min after oral administration which meant that it had a rapid and wide distribution. The highest tissue concentration was found in stomach and was much higher than in all the other organs, which was relative to the route of administration and the acidic nature of Sal A. Besides the gastrointestinal tract, Sal A had a relative high distribution in liver, followed by heart, lung and kidney, which implied that the distribution of Sal A depended on the blood flow or perfusion rate of the organ. In addition, Sal A could also be detected in brain homogenate, suggesting that it could efficiently cross the blood–brain barrier which provided a basis for the curative effect against peripheral neurological dysfunction in diabetic rats (Yu et al. 2012).

### Excretion of Sal A from rats

**Figure 5.**
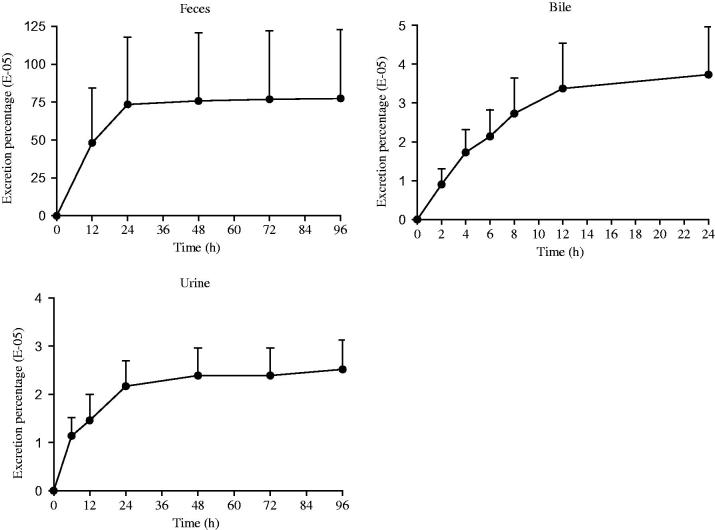
Excretion–time curves of Sal A following a single-dose oral administration of 20 mg/kg to rats (*n*= 6).

After a single oral administration of Sal A (20 mg/kg) to intact rats, the major route of elimination of the compound was via feces (a mean of 0.775% of the dose). Bile elimination was minor (a mean of 0.00373%) followed by urinary excretion (a mean of 0.00252%). Results were shown in Figure 5. The elimination of prototype drug was rapid in both sexes, but the recovery was less than 1% of the administered dose which indicated that a large proportion of Sal A must be metabolized or transformed *in vivo*.

## Discussion and conclusions

In the current study, the absorption, distribution and elimination of Sal A after a single oral administration at doses ranging from 5 to 20 mg/kg have been fully characterized in rats.

After taken orally, Sal A was absorbed rapidly in rats (*T*_max_=1 h), which was consist with the former report (Pei et al. 2008). The total exposure increased linearly over the dose range of 5–20 mg/kg, and the peak exposure was directly related to the dose. A short *t*_1/2_ (1.72–1.96 h) indicated that Sal A was rapidly eliminated from the body at experimental doses and the similar absorption trend was also found in our previous Beagle dog study (Sun et al. 2013). The plasma half-life was much longer in the intravenous experiment (6.16 h) and the pharmacokinetic study after oral administration at the dose of 100 mg/kg (3.29 h) (Pei et al. 2008). The explanation was that the elimination rate of Sal A in organism was limited and the metabolic enzyme activity was saturated.

The mean plasma concentration–time curve of Sal A after oral administration showed significant double peaks which was mainly caused by the enterohepatic circulation. Besides, the results of tissue distribution showed that Sal A had an extensive and high distribution. Re-release from tissues might also lead to the increase of plasma concentration and result in the multimodal phenomenon.

The absolute bioavailability of Sal A administered by the oral route in pure form was calculated to be 0.39–0.52%, which was less than the value reported by Lai et al. (2011) (2.5%). Based on these results, we speculated that salvianolic acid B and rosmarinic acid might promote the absorption of Sal A. The MRT and *t*_1/2_ in both experiments were consistent, indicating that the two compounds might not affect the clearance of Sal A. Compared with our former study in Beagle dogs (Sun et al. 2013), no species difference was observed. The instability of Sal A in the intestinal alkaline environment (Liu et al. 2011) and the first-pass metabolism might contribute to its low absorption. Based on our results in rats and Beagle dogs, we speculate that the bioavailability of Sal A in humans should be relatively low. However, it is known that the metabolizing enzymes of the test system (rat and dog) and human are quite different and distinct. Therefore, experiments performed in monkeys that are closest to human should be conducted later to obtain more credible results. In addition, the metabolic differences between rats and humans will be evaluated through an *in vitro* liver microsomal incubation system in our subsequent studies.

Results of permeability study in Caco-2 monolayer showed that *P*_app_ value of Sal A was less than 1 × 1 0^−6^ cm/s, which meant that Sal A had a poor permeability. The preceding Caco-2 data suggested that the gastrointestinal absorption of Sal A was characterized by passive diffusion (Lu et al. 2008). However, the ratio of the *P*_app_ values of basolateral to apical and apical to basolateral were about 3 in our experiment, indicating that the transport of Sal A across the Caco-2 monolayer involved an active transport mechanism. This was probably caused by the extremely low transport and the low concentrations measured. Besides, the instability of Sal A in the partial neutral environment of Hanks and the lack of stabilizer could also influence the result. Caco-2 cells had high expression levels of the multidrug transporters P-gp and MRP2. The active transport mechanism involved in the transportation of Sal A remains to be done in the future.

The accumulated excretion percentage of Sal A through feces, bile and urine was less than 1% of dosage. Five metabolites of Sal A were identified as Sal A-monoglucuronide, monomethyl-Sal A-monoglucuronide, mono-methyl-Sal A, dimethyl-Sal A and dimethyl-Sal A-monoglucuronide by Shen et al. (2009) in plasma, which indicated two metabolic pathways (methylation and glucuronidation) of Sal A *in vivo*. Han et al. (2012) further figured out two major isoforms of UGTs, UGT1A1 and UGT1A9, that catalyzed the glucuronidation of Sal A. Besides phase II drug metabolism, Sal A was found instable in distilled water even at room temperature (Xu et al. 2008). Xu et al. (2011) isolated and identified six different degradation products by NMR and LC–MS analysis, including four mono-oxygenation products and two dehydrogenation products. According to our result and the former reports, the majority of the administered drug was degraded into its oxidation forms or metabolized to be its glucuronidation and methylation products *in vivo*.

Previous pharmacological studies in our laboratory found that Sal A exerted beneficial effects after oral administration on plantar microcirculation and peripheral neurological dysfunction, and could protect against vascular endothelial dysfunction in diabetic rats (Yang et al. 2011a; Yang et al. 2011b; Yu et al. 2012; Hou et al. 2017). Pharmacological experiments showed that Sal A exerted curative effect in glucose consumption experiment and insulin collaborative experiment *in vitro* at the concentration of 1 0^−10^–1 0^−6^ mol/L. Results of our experiment showed that Sal A could be detected in brain homogenate, which provided a basis for the curative effect against peripheral neurological dysfunction in diabetic rats. Furthermore, after oral administration of Sal A at the similar dosage as applied in pharmacodynamic experiments *in vivo*, concentration of Sal A could reach the effective level as *in vitro*, which provided evidence for its therapeutic effects on diabetic complications after oral administration.

In conclusion, this study was the first to systematically characterize the pharmacokinetic properties of Sal A after oral administration in rats and the results could support its future development as an oral drug for the treatment of diabetic complications. Further study of its interaction with other substances is of significance for improving its bioavailability. In addition, the role of drug transporters and first-pass metabolism in the low bioavailability of Sal A and the metabolic differences between rats and humans remain to be studied in the future.
